# Hydroxide-rich polypropylene hybrid microparticles for the removal of reactive yellow 145 from aqueous solutions: equilibrium, kinetic and thermodynamic studies

**DOI:** 10.1186/s13065-025-01517-y

**Published:** 2025-05-30

**Authors:** Ahmed Bakry, Salwa M. Elmesallamy

**Affiliations:** 1 Chemistry Department, Faculty of Science, Helwan University, Ain Helwan 11795, Cairo, Egypt; 2 Polymer Laboratory, Petrochemical Department, Egyptian Petroleum Research Institute, Naser City, Cairo, Egypt

**Keywords:** Polypropylene, Reactive yellow 145, Metal hydroxide, Adsorption, Kinetics, Thermodynamics

## Abstract

Cleaning wastewater from harmful dyes is mandatory to keep the safety of humans and other lives forms. In this study, hybrid polypropylene/metal hydroxide microparticles were presented for the removal of reactive yellow 145 (RY145) from aqueous solutions. The chemical structure and morphological features of the adsorbent were studied by Fourier transform infrared (FT-IR), energy dispersive X-ray spectroscopy (EDS) and scanning electron microscopy (SEM). To achieve high adsorption efficiency, the adsorption process was optimized using different adsorbent doses, pH of the medium, time intervals and temperature. Kinetic and isotherm models were utilized to analyze the adsorption data. The pseudo-second order and Langmuir models showed the best fit for the adsorption process. A maximum adsorption capacity of 39.62 mg g^−1^ was attained at 25 °C and neutral pH. The thermodynamic parameters of the adsorption process were estimated. The adsorption process increased by increasing temperature revealing that the adsorption is endothermic (∆ *H* =  25.52 kJ mol^−1^ K^−1^) but random (∆*S* =  90.61 J mol^−1^ K^−1^) and feasible (∆*G* =  −  1.48 kJ mol^−1^). Hence, it can be concluded that the adsorption process was endothermic but random and feasible.

**Graphical Abstract:**

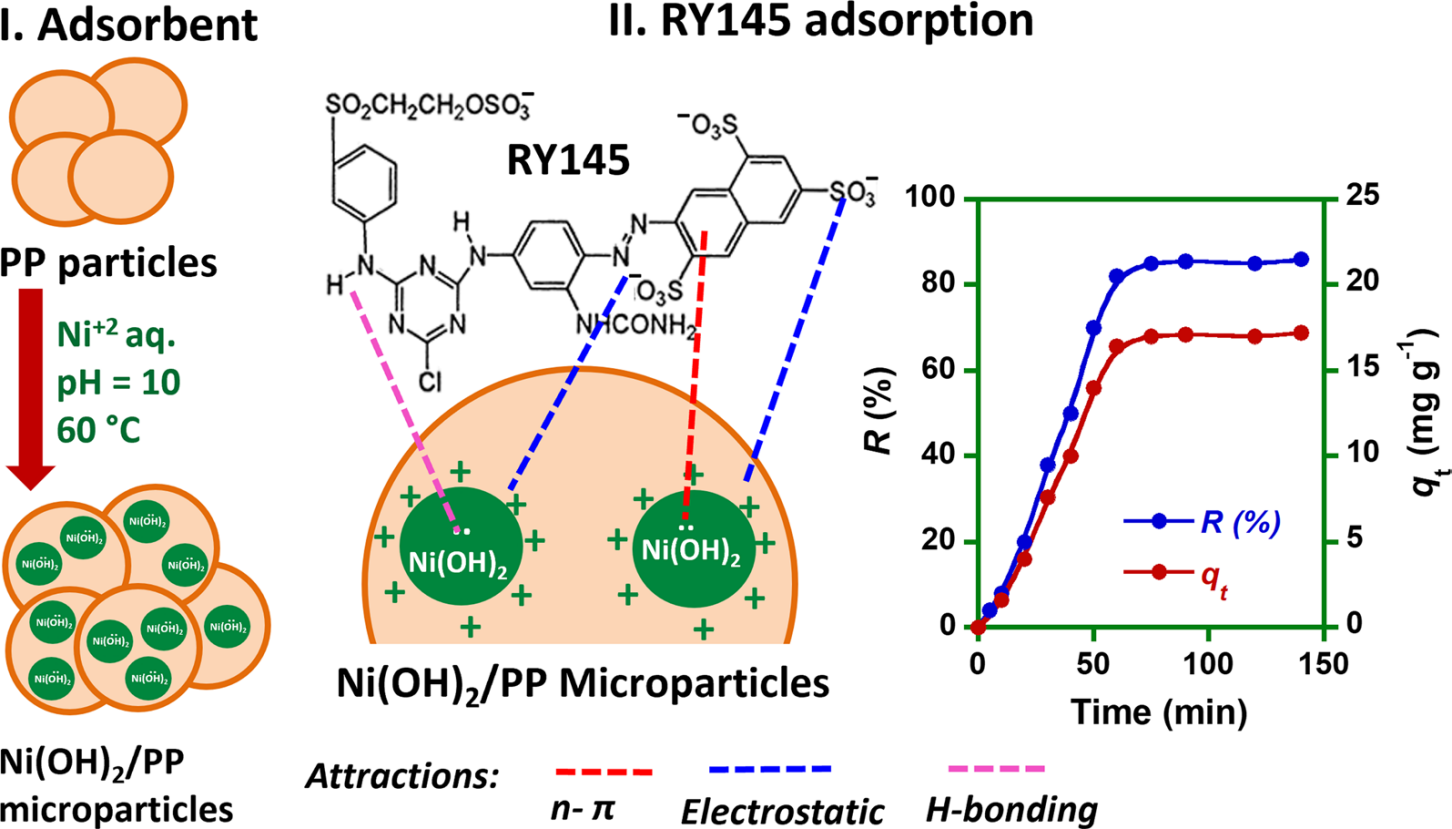

## Introduction

Rapid population growth and industrialization have led to environmental pollution that negatively affects people’s health. For example, water sources are continuously polluted due to excessive discharge of dyed effluents from various industries such as textile, rubber, pharmaceutical, and cosmetic industries [1]. These dyes exhibit stable molecular structures that resist biodegradation and decomposition by oxidants agents [1]. Dye impurities in water diminish water transparency and aeration, and thus decrease photosynthetic processes and dissolved oxygen content in water. In addition, the synthetic dyes are usually toxic and carcinogenic [2]. To circumvent this environmental issue, dyed wastewater has been purified using various technologies such as membrane separation [3], ion-exchange [4], and adsorption [5, 6]. Due to its simplicity, flexibility, low cost, reusability, and high efficacy, adsorption is the most economical and reliable process [7]. Various adsorbents were investigated to remove harmful dyes from water including polymers [5, 6], clays [8], fly ash [9], and activated carbon [10].

Polymers offer large surface area to volume ratios, high mechanical properties [11, 12], adjustable surface chemistry and pore size distribution [13], and ease of polymer regeneration and dye recovery [14]. Although polymers are outstanding and applicable materials, it is sometimes necessary to treat these polymers before they are ready for a particular application [15–18]. For example, polystyrene was sulfonated [19] or endowed with hydroxide groups for attaining valuable adsorbents to uptake dyes from contaminated water [20].

Polypropylene has many useful properties such as mechanical and thermal stability. However, the high surface hydrophobicity and the lack of polar groups on the surface could limit the applications of the polymer [21]. Accordingly, the use of polypropylene as an adsorbent has not been sufficiently investigated in literature. Nevertheless, various approaches have been used to provide the surface of polypropylene with suitable functional groups. Examples of these approaches are dielectric barrier discharge plasma treatment in air [21] and chemical surface treatments with nitric acid [6] or sulfuric acid [5, 22].

Polymer hybrid materials have better properties than single polymers [23, 24]. For instance, the combination of inorganic micro/nanoparticles (e.g., ion exchangers, metal oxides, zero valent Fe), as effective adsorption sites, with polymers produces efficient polymer/inorganic hybrid adsorbents for water remediation [20, 25]. Inorganic particles exhibit a high tendency toward pollutants in waters, but their tendency to aggregate and low mechanical properties make them useless on their own [26, 27], so they are combined with supporting materials such as polymers [28, 29]. For instance, polymers/hydroxides hybrids have been introduced for the efficient uptake of toxic organic dyes from wastewater [30]. The hydroxide-containing hybrids exhibit large surface area, chemical stability, non-toxicity, cost-effective, availability and regeneration ability, and thus offer outstanding adsorption performance.

Reactive yellow 145 (RY145 dye) is frequently used for dyeing cotton, rayon and polyesters in the textile industry, in printing and in tanneries. However, the adsorption of RY145 has not been adequately studied, although it has been associated with mutagenic and carcinogenic effects on humans and aquatic organisms. It has both an azo group and a sulfate group, which makes it recalcitrant by nature [31]. Moreover, low adsorption capacities are achieved with adsorbents such as teff straw-activated carbon (17 mg g^−1^) [32] and biochar derived from groundnut Shel (7.3 mg g^−1^) [33].

The aim of this work was therefore to develop a valuable polypropylene hybrid adsorbent for the uptake of reactive yellow 145 (RY145 dye) from aqueous solutions. PP microparticles were loaded with nickel hydroxide to provide their surface with hydroxide groups. It was assumed that the hydroxide groups could serve as binding sites for RY145 dye. The adsorption behavior of RY145 dye on the nickel hydroxide loaded PP microparticles (Ni/PP) was investigated using batch experiments under different operating parameters (adsorbent dosage, system pH, and retention time). Kinetic models (the pseudo-first-order and pseudo-second-order models), and isotherms (Langmuir and Freundlich) were utilized to define the models that best fit the experimental results. The thermodynamic parameters: Enthalpy (Δ*H*), Gibbs free energy (Δ*G*) and entropy (Δ*S*) of the adsorption process were calculated. According to the best of our knowledge, this is the first comprehensive study about exploiting the capability of hydroxide-containing PP microparticles to remove reactive dyes from water.

## Experimental

### Materials

Polypropylene powder (Accurel EP-100, 200–400 µm) was a gift from Akzo (Obernburg, Germany). Nickel (II) chloride hexahydrate, sodium hydroxide, and hydrochloric acid were received from El-Gomhoria Co., Cairo, Egypt. RY145 was supplied from Burboya (Bursa, Turkey), which has physicochemical properties as reported in Table 1.

**Table 1:** Physical and chemical properties of RY145 dye

C.I. generic name	Reactive yellow RY 145 dye
Chemical class	Azo dye – vinyl sulfone type
Chemical structure	
CAS registry number	93,050–80-7
Molecular formula	C28H20ClN9Na4O16S5
Molecular weight	1026.25
Physical state	Powder
λ_max_	417 nm
Solubility in water at 30 °C	80 g L^−1^

### Preparation of NiH/PP hybrid adsorbent

PP particles were mixed with an aqueous solution of NiCl_2_.6H_2_O (0.01 M) preheated at 60 °C. The mixing was continued for 0.5 h followed by adding drops of an aqueous sodium hydroxide solution (0.1 M) to raise the medium pH to 10 till precipitating the metal hydroxide on the polymer microparticles. After 1 h, NiH/PP microparticles were gathered through filtration. The sample was washed several times with deionized water. Nickel hydroxide loaded PP microparticles (NiH/PP) were dried in an oven at 50 °C. The amount of metal hydroxide loaded on the surface of NiH/PP was calculated by subtracting the mass of PP particles originally used during the reaction from the final mass corresponding to NiH/PP particles. A value of 0.27 g Ni(OH)_2_ per gram NiH/PP microparticles was found.

### Characterization of NiH/PP microparticles

The structural features of NiH/PP microparticles were analyzed by Fourier-transform infrared (FT-IR) spectroscopy utilizing a Perkin-Elmer Spectrometer 400 (PerkinElmer Inc., Waltham, MA, USA). The analysis was performed utilizing a Golden Gate diamond single reflection device. The measurements were carried out using the range 4000 to 400 cm^−1^ and a resolution of 2 cm^−1^. The morphological features of NiH/PP samples were examined using a QUANTA FEG 250, USA scanning electron microscope (SEM). NiH/PP microparticles were spread on a double sides adhesive tape attached on SEM specimen holder. Images were acquired using an accelerating voltage of 20 kV and at different magnifications. The quantitative elemental analysis was done utilizing the energy—dispersive X-ray (EDX) unit attached to the SEM instrument. The average particle size and zeta potential were analyzed using a Malvern Zetasizer (Ver. 6.32, Malvern Instruments, Ltd.). For zeta potentials measurements, samples were equilibrated in KCl electrolyte solution (0.001 M) for 2 h at 25 °C before measurements. The textural properties (specific surface area, pore volume, and pore size) were investigated by N_2_ sorption–desorption measurements using a Quantachrome TouchWin™ version 1.21 instrument. The specific surface area (S_BET_) was calculated using the Brunauer–Emmett–teller (BET) equation. The pore volume (V_T_) and pore size (D) were calculated using the Barrett–Joyner–Halenda (BJH) method.

### RY145 dye adsorption

The adsorption of RY145 dye on NiH/PP microparticles was studied using batch technique. A stock solution of RY145 dye (100 mg L^−1^) in distilled water was prepared, and other adsorbate solutions having lower concentrations were obtained by suitable dilution.

For the kinetic studies, adsorption was investigated at different reaction times (10–140 min). 40 mg NiH/PP microparticles were shaken with 40 ml of the dye solution (20 mg L^−1^, pH 6.8) in a thermoshaking water bath at 25 °C for a certain time.

The effect of the polymer dose was investigated by mixing different amounts of NiH/PP microparticles (0.25—4 g L^−1^) with 40 ml of the dye solution (20 mg L^−1^, pH 6.8) for a period of 140 min at 25 °C.

To determine the influence of the pH value of the dye solution, pH values between 2 and 12 were used. The pH of the solution was adjusted with 0.1 M HCl and 0.1 M NaOH. NiH/PP microparticles (1 g L^−1^) were mixed with the dye solution (20 mg L^−1^) for 140 min at 25 °C.

Equilibrium studies were performed using different initial dye concentrations (10—40 mg L^−1^, pH 6.8) and 1 g L^−1^ NiH/PP microparticles at 25 °C for 140 min.

For thermodynamic studies, the adsorption process was carried out at different temperatures (15—45 °C), a dye solution concentration of 20 mg L^−1^ and an adsorbent dose of 1 g L^−1^ for 140 min.

After each adsorption test, the mixture was filtered through a 0.45 μm membrane. The absorbance of the dye solution was measured utilizing an UV − visible spectrophotometer (Jasco V-550, Japan) at 417 nm. The concentration of the dye was then derived from a previously prepared calibration curve for the dye solutions with specific concentrations. Each adsorption experiment was repeated 5 times, and the results were reported as the mean value ± SD. Both dye removal efficiency (*R*, %) and the adsorption capacity (*q*_e_) of the NiH/PP microparticles were estimated using Eq. 1 and Eq. 2, respectively.

1 $$R \left( {\text{\% }} \right) = 100 x \left( {1 - \frac{{C_{{\text{e}}} }}{{C_{{\text{o}}} }} } \right)$$

2 $$q_{{\text{e}}} \, = \,(C_{{\text{o}}} - C_{{\text{e}}} ) x \frac{V}{m}$$

where *C*_o_ and *C*_e_ are the initial and equilibrium dye concentrations, respectively, (mg L^−1^); *q*_e_ is mass of the adsorbed dye per gram adsorbent (mg g^−1^); *V* is the volume of dye solution (L); *m* (g) is the adsorbent’s mass.

### Adsorption kinetic analysis

To explore the rate and mechanism of adsorption, non-linear kinetic models of pseudo-first order (Eq. 3) [34], pseudo-second-order (Eq. 4) [35], intraparticle diffusion (Eq. 5) [36] and Elovich (Eq. 6) [37] were tested to fit the experimental data of the adsorption process.

3 $$q_{t} = q_{e} \left( {1 - e^{{ - k_{1} t}} } \right)$$

4 $$q_{t} = \frac{{q_{e}^{2} k_{2} t}}{{1 + k_{2} q_{e} t}}$$

5 $$q_{t} = k_{i} t^{0.5} + C_{i}$$

6 $$q_{t} = \left( {\frac{1}{\beta }} \right)\ln \left( {1 + \alpha \beta t} \right)$$

where *q*_t_ and *q*_e_ (mg g^−1^) are the adsorption capacity at time *t* and equilibrium, respectively; *k*_1_ and *k*_2_ (min^−1^) are the adsorption rate constants of pseudo-first-order, and pseudo-second-order models, respectively; *K*_i_ is the intraparticle diffusion rate constant (mg g^−1^ min^0.5^); *C*_i_ is the boundary layer thickness; *α* is the initial adsorption rate (mg g^−1^ min^–1^); *β* is the desorption constant (g mg^−1^).

### Adsorption isotherm analysis

Various adsorption isotherms were applied to the experimental results obtained at 25 °C: Langmuir (Eq. 7), Freundlich (Eq. 8) [38], Dubinin–Radushkevich isotherm models (Eqs. 9–11) [39], and Temkin (Eqs. 12 and 13) [40].

7 $$q_{e} = \frac{{q_{m} k_{L} C_{e} }}{{1 + k_{L} C_{e} }}$$

8 $$q_{e} = K_{F} C_{e}^{{1/n_{F} }}$$

9 $$q_{e} = q_{DR} exp\left( { - K_{DR} \varepsilon^{2} } \right)$$

10 $$\varepsilon = RTln\left( {1 + \frac{1}{{C_{e} }}} \right)$$

11 $$E = \frac{1}{{\sqrt {2K_{DR} } }}$$

12 $$q_{e} = Bln\left( {A_{T} C_{e} } \right)$$

13 $$B = \frac{RT}{{b_{T} }}$$

where *C*_e_ (mg L^−1^) and *q*_e_ (mg g^−1^) are the aqueous concentration of the dye and the amount of dye taken by the adsorbent, respectively, when equilibrium reaches; *q*_max_ is the maximum adsorption capacity of the adsorbent; *K*_L_ is the Langmuir constant, which indicates the energy of adsorption and affinity of the adsorbing positions (L mg^−1^); *n* and *K*_f_ [mg g^−1^ (L mg^−1^)^n^] are the Freundlich constants, which reflect the intensity and capacity of adsorption, respectively. *q*_DR_ is a constant in the Dubinin-Radushkevich isotherm model which is related to adsorption capacity; *ε* is Polanyi potential; *K*_DR_ (mol^2^ kJ^−2^) is Dubinin-Radushkevich constant related to the mean free energy of adsorption; *R* is gas constant (J mol^−1^ K^−1^), *T* is absolute temperature, and *E* is mean adsorption energy; B is a constant related to heat of sorption(J/mol), A_T_ is the Temkin isotherm equilibrium binding constant (L g^−1^), b_T_ is the Temkin isotherm constant. The Langmuir model assumes that a certain number of adsorbing sites exist on the sorbent in homogeneous distribution and with similar adsorption affinity. The Langmuir model is useful to deduce the maximum adsorption capacity of the sorbent, which cannot be known experimentally. The Freundlich adsorption isotherm supposes that the adsorbing locations on the sorbent have different affinities for the sorbate and that it is possible for several adsorbate layers to form on the sorbent surface [41]. This model assumes that the more active binding sites are initially occupied than less ones and that the binding strength decreases as the sites are progressively occupied by the adsorbate.

### Regeneration and reusability studies

Reusability of the dye loaded adsorbent microparticles was tested by shaking them with distilled water (250 mg in 50 ml) at an initial pH of 9 for 24 h. pH of 9 was attained by adding proper extents of NaOH (0.01 M) to distilled water. The microparticles were collected, washed with distilled water and dried before being used for the subsequent adsorption–desorption cycle. The adsorption efficiency was determined for five cycles of adsorption–desorption experiments and then compared.

## Results and discussions

### NiH/PP microparticles characterization

**Fig. 1:**
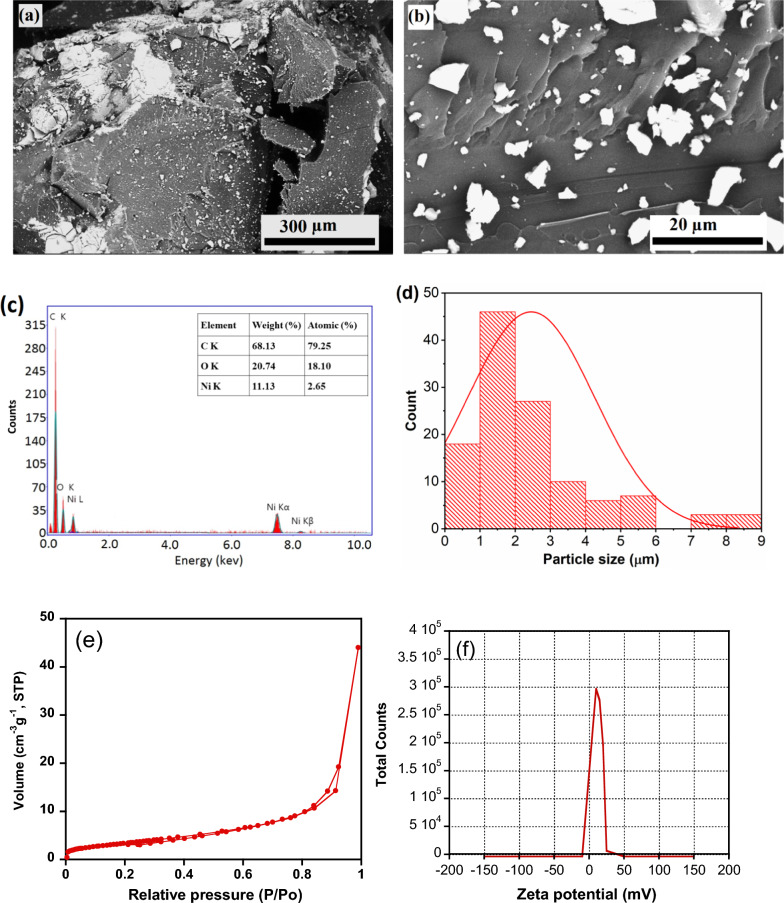
Characteristics of NiH/PP microparticles: (**a**) and (**b**) SEM images at different magnifications, (**c**) EDS profile of C, Ni, and O elements, (**d**) the particle size distribution of Ni(OH)_2_ particles on NiH/PP microparticles, (**e**) N_2_ sorption–desorption curves, and (**f**) Zeta potential

The morphology studies of the NiH/PP microparticles (Fig. 1 a and b) exhibit the appearance of nickel hydroxide particles on the surface of PP particles. Ni(OH)_2_ particles (the white spots) seems to distribute well on PP microparticles. The Ni and O from the metal hydroxide were detected on NiH/PP microparticles by EDS analysis as illustrated in Fig. 1c; elemental analysis results are given in the inset. The peaks related to Ni, O and C in the EDS spectrum confirm the development of NiOC phase on NiH/PP microparticles. The particle size distribution of Ni(OH)_2_ particles is illustrated in Fig. 1d, where they exhibit a small lateral size of 2.5 ± 0.6 µm. The surface area and pore structure of microparticles were measured because of their effect on the adsorption capacity of the sorbent [42]. Proper surface area, porous structure, and extents of active sites are necessary to attain efficient adsorption processes [43]. The results of the BET analysis (Fig. 1e) show that the adsorbent has specific surface area value of 16.45 (m^2^ g^− 1^), total pore volume equals 0.071 (cm^3^ g^− 1^) and average pore radius of 5.5 nm. A pore diameters less than 50 nm, revealing that the samples have mesoporous structures (pore size 2—50 nm) which favor adsorption. The zeta potential of the microparticles was measured at neutral pH (Fig. 1f). Microparticles exhibited a zeta potential value of + 9.4 mV.

**Fig. 2:**
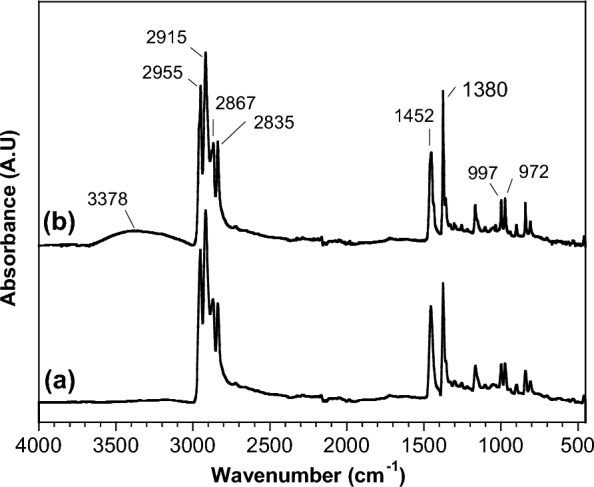
FT-IR spectra for (**a**) PP and (**b**) NiH/PP microparticles

The surface chemistry of PP microparticles before and after loading Ni(OH)_2_ on the surface was analyzed by FT-IR analysis (Fig. 2). The spectra of the samples show the main characteristic peaks of PP molecular structure: at 2876 cm^−1^ and 2955 cm^−1^ (– CH_3_ stretching vibration), 2835 and 2915 cm^−1^ (– CH_2_ stretching vibration), 1452 cm^−1^ and 1380 cm^−1^ (– CH_2_ bending vibration). After the deposition of Ni(OH)_2_ on PP microparticles, a new broad band centered at 3378 cm^−1^ was observed (Fig. 2b). This band is assigned to the stretching vibration of the – OH groups from Ni(OH)_2_ phases present on NiH/PP microparticles. Hence, SEM, EDS and FT-IR observations confirm the precipitation of Ni(OH)_2_ particles on PP microparticles.

### Adsorption results

#### Effect of pH

**Fig. 3:**
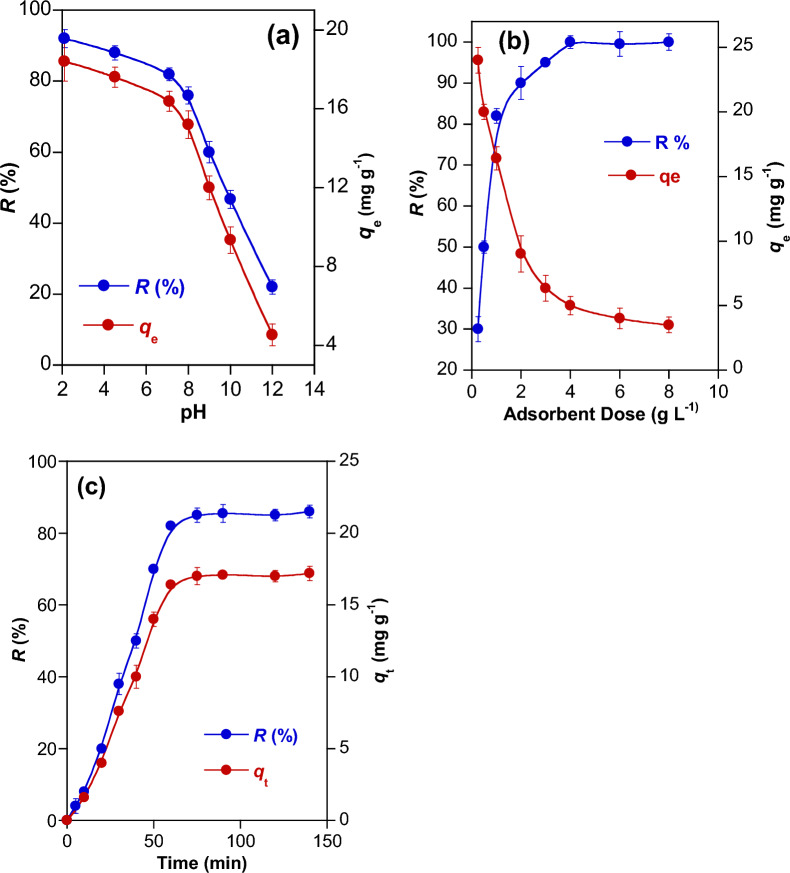
Influence of various parameters on the RY145 dye removal (*R*, %) and adsorption capacity (*q*_e_) of NiH/PP microparticles at 25 °C: (**a**) pH of the medium, (**b**) dose of the adsorbent, and (**c**) contact time

The variations in *R* (%) and *q*_e_ values of with the variation of pH, adsorbent dose and time of contact time is reported in Fig. 3a–c. The medium pH influences the state of the surface charges on the sorbents. This in turn affects the intensity of the electrostatic attractions that take place at the adsorbate/adsorbent interface. The influence of pH on the adsorption of RY145 dye on NiH/PP microparticles was examined in the pH range 2–12 (Fig. 3a). The values of *R* % and *q*_e_ tend to decrease with enhancing the medium pH. The greatest values of *R* % (92%) and *q*_e_ (21 mg g^−1^) were observed at pH 2, and the lowest ones at pH 12 (*R* % = 22% and *q*_e_ = 4.54 mg g^−1^). These observations infer that the adsorption of RY 145 dye on NiH/PP microparticles intensely depends on the variations in the surface charge of the metal hydroxide caused by the alteration of the medium pH, as shown in Eq. 14 [44].

14 $${\mathbf{Ni}}^{2 + } \mathop \leftarrow \limits^{{ + \user2{ H}^{ + } }} \left[ {{\mathbf{Ni}}\left( {{\mathbf{OH}}} \right)_{1} ]^{ + 1} \mathop \leftarrow \limits^{{ + \user2{ H}^{ + } }} } \right[{\mathbf{Ni}}\left( {{\mathbf{OH}}} \right)_{2} ]^{0} \left( {\varvec{s}} \right)\to ^{{ + \user2{ OH}^{ - } }} \user2{ }[{\mathbf{Ni}}\left( {{\mathbf{OH}}} \right)_{3} ]^{ - 1}$$

It is known that the surface of metal hydroxide at low pH acquires positive charges because of the uptake of H^+^ ions, which favors the interaction with anionic species. Increasing the pH in basic media decreases the positive charges on the surface of metal hydroxide because OH¯ ions tend to attach to the surface and form hydroxo-complex anions [M(OH)_n+1_]¯ that hinder the uptake of anionic species. Hence, the efficacy of adsorption decreased significantly in basic media. These observations indicate that adsorption occurs through the electrostatic attractions between the sulfonic groups (—SO3¯) having negative charges on RY 145 dye and the positive charges on the NiH/PP microparticles. Similar observations were reported when reactive dyes were adsorbed from water on metal hydroxide sludge [44]. Measurements of pH of the dye-composite mixture after adsorption showed a slight increase in pH. For example, in adsorption experiments conducted at an initial pH of 6, the final pH was 6.8. This behavior was previously justified by the release of hydroxyl ions from the metal hydroxide, which were replaced by the strong—SO3¯ cation exchange groups on the dye [44]. This implies RY145 dye could also be adsorbed on NiH/PP hybrid adsorbent by ion exchange to maintain a neutral charge.

#### Effect of adsorbent dose

The influence of NiH/PP microparticles dose (0.25 – 4 g L^−1^) on the adsorption of RY145 dye at 25 °C and neutral pH for one hour is given in Fig. 3b. The removal efficiency increases considerably with enhancing dose of the adsorbent from 0.25 g L^−1^ (*R* = 30%) to 1 g L^−1^ (*R* = 82%). This is because increasing the adsorbent dose provides more available binding sites for attracting the pollutants from water, thus the removal efficiency. Additional enlargement in the adsorbent dose from 1 to 4 g L^−1^ also resulted in an increase in *R* values, but to a lesser extent. Complete removal of the dye was achieved when 4 g L^−1^ or higher doses of the adsorbent were used. The adsorption capacity (*q*_e_) showed an indirect dependence on the dose of the adsorbent. It decreased by half when the adsorbent dose increased from 0.25 g L^−1^ to 4 g L^−1^. This can be attributed to the existence of excess adsorption sites on NiH/PP microparticles that could not be occupied by the molecules of RY145 dye, as reported by others [5].

#### Effect of contact time

The temporal changes of *R* (%) and *q*_e_ that occurred during adsorption were followed (Fig. 3c). Adsorption was rapid throughout the first hour, reaching a removal of *R* = 82%. With longer time of contact, the adsorption slowed down until equilibrium was reached. At the beginning of the adsorption, there are many available adsorption locations for removing adsorbate, which allows for rapid adsorption. As soon as these locations become almost completely occupied by the adsorbate, the inclination of the sorbent to the adsorbate also decreases, so that the adsorption process slows down.

#### Kinetic studies

**Fig. 4:**
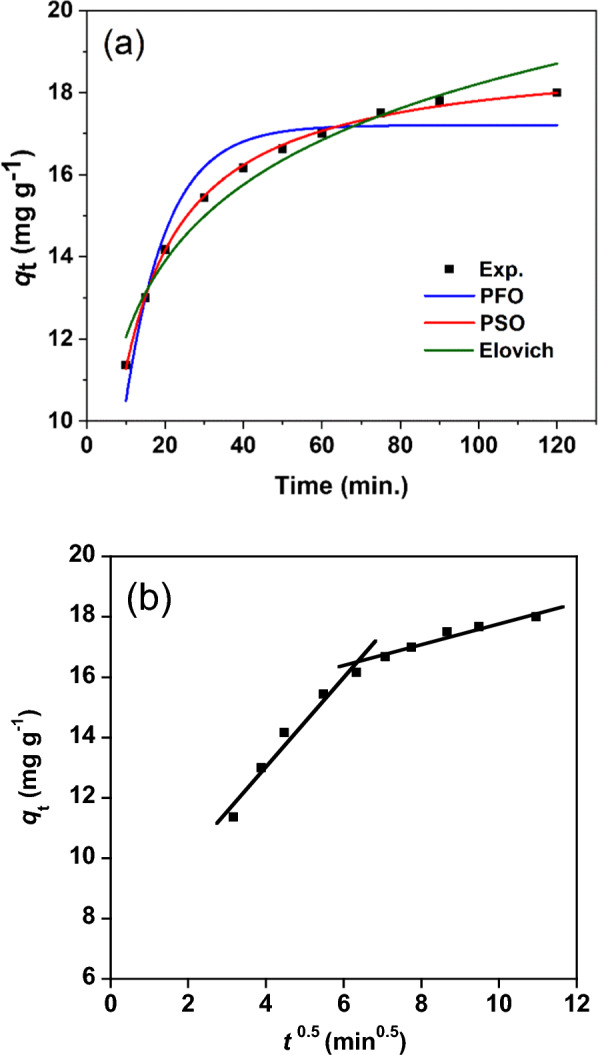
Kinetic fits for adsorption of RY 145 dye on NiH/PP microparticles using (**a**) pseudo-first-order (PFO), pseudo-second-order (PSO), and Elovich kinetics models and (**b**) intra-particle diffusion model

The kinetics of adsorption were studied to investigate the mechanism of adsorption, equilibrium time and the rate limiting steps. Experimental data of RY145 dye adsorption on NiH/PP microparticles were fitted using the kinetic models (Eqs. 3-6) as introduced in Fig. 4. The fitting parameters for the tested kinetic models and the corresponding correlation coefficients (*R*^2^) are given in Table 2. A higher *R*^2^ value and a calculated adsorption capacity (*q*_e,cal_) closer to the experimental capacity (*q*_e,exp_) were observed when the pseudo-second order was used to fit the data than in the case of utilizing the pseudo-first order. Thus, the pseudo-second order showed the best correlation of the adsorption kinetics. Similar kinetic profile was reported for the adsorption of RY145 dye on iron oxide microparticles with fern leaf morphology [45] as well as chitosan Functionalized by Talc and Cloisite 30B [46]. Intraparticle diffusion, which possibly occurred during the adsorption process, was investigated using the kinetic model of Weber and Morris (Eq. 5). This model provides the mechanism and rate-limiting step of the adsorption process by examining the correlation between the extent of adsorption and the square root of the contact time (*t*^0.5^) instead of (*t*). When a plot of qt versus *t*^0.5^ gives a straight line through the origin, intraparticle diffusion is the only rate-controlling step [47]. In this case, two intersecting lines were noted (Fig. 4b), which reveals that the adsorption process occurred through two controlling steps, namely surface adsorption and intraparticle diffusion [48]. Surface adsorption took place at *t*^0.5^ below 6.45 min^0.5^, followed by intraparticle diffusion at larger values of *t*^0.5^. The rate constants for surface adsorption (*k*_i1_) and intraparticle diffusion (*k*_i2_) are reported in Table 2. Intraparticle diffusion was not the only rate-determining step, although it had a lower rate than surface adsorption (*k*_i2_ < *k*_i1_). These observations reveal that the adsorption occurred in the first stage on the surface of the adsorbent (high rate), and occurred in the second stage through the pores of the adsorbent.

**Table 2:** The values of fitting parameters of adsorption kinetics models of RY145 dye on the NiH/PP microparticles

*q*_e,exp_	Models	Model Parameters
18.20 mg g^−1^	pseudo-first-order	*q*_e,cal_ = 17.12 (mg g^−1^) *k*_1_ = 0.0941 (min^−1^) *R*^2^ = 0. 9278
	pseudo-second-order	*q*_e,cal_ = 18.98 (mg g^−1^) *k*_2_ = 0.0076 (g mg^–1^ min^–1^) *R*^2^ = 0.99882
	Elovich	*α* = 23.34 mg g^−1^ min^–1^ *β* = 0.371 g mg^−1^ *R*^2^ = 0.96498
	Intra-particle diffusion	*k*_i1_ = 1.495 mg g^−1^ min^0.5^ *C*_*1*_ = 7.08 *R*^2^ = 0.96327 *k*_i2_ = 0.339 mg g^−1^ min^0.5^ *C*_*2*_ = 14.44 *R*^2^ = 0.9486

#### Adsorption isotherm

**Fig. 5:**
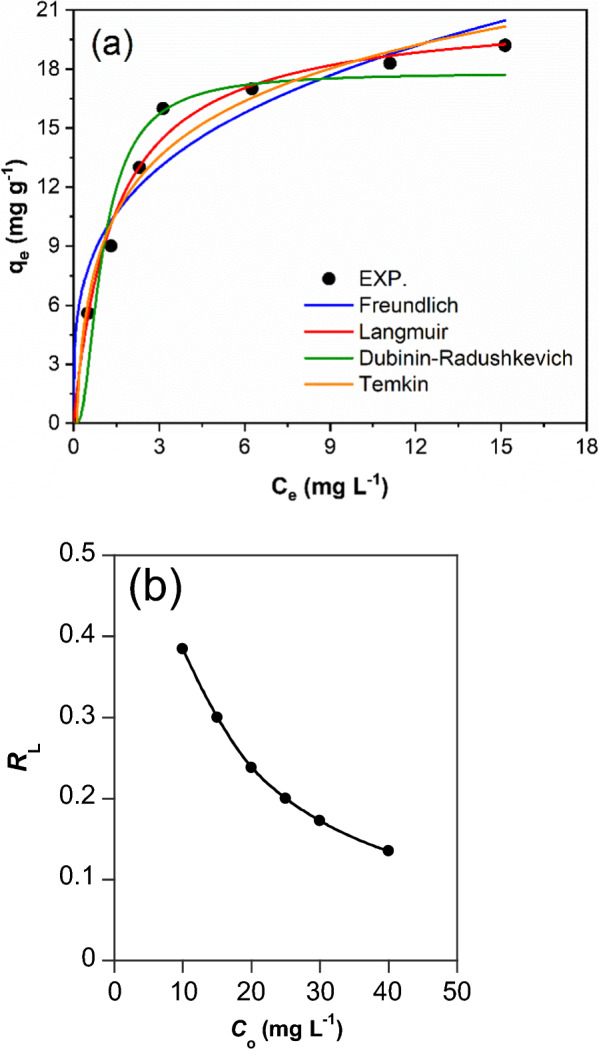
(**a**) Isotherm model plots of Freundlich, Langmuir, Dubinin–Radushkevich, and Temkin for the adsorption of RY145 dye adsorption onto NiH/PP microparticles; (**b**) the equilibrium parameter (*R*_L_)

The equilibrium of the adsorption was studied utilizing various dye solutions. Both Langmuir (Eq. 7) and Freundlich adsorption isotherms (Eq. 8) were applied to the data of the adsorption process (Fig. 5) and the parameters of isotherms are summarized in Table 3. The Langmuir adsorption isotherm showed a greater regression coefficient (*R*^2^) than that shown by Freundlich isotherm. Therefore, the Langmuir isotherm (*R*^2^ > 0.99) better defines the adsorption behavior than the Freundlich isotherm. This discloses that the adsorption of RY145 dye on NiH/PP microparticles is a homogeneous and a monolayer process. The same isotherm performance was reported for water treatment using sunflower husks biochar [49] and chitosan functionalized talc [46]. The maximum adsorption capacity (*q*_max_), estimated by Langmuir isotherm, of NiH/PP microparticles is 39.62﻿ mg g^−1^. The relevance of the value of *q*_max_ observed for NiH/PP microparticles compared with other values reported by others is given in Table 4. NiH/PP microparticles shows a good removal efficiency at 25 °C, which signifies that the adsorption process is not an energy consuming process. The parameter *K*_L_ and *C*_o_ of the dye solution can be further used to evaluate the viability of the adsorption process by calculating the equilibrium parameter (*R*_L_) utilizing Eq. 15.

15 $$R_{L} = \frac{1}{{1 + K_{L} C_{o} }}$$

**Table 3:** The constants of the Langmuir, Freundlich,  Temkin, and Dubinin-Radushkevich adsorption isotherms

Models	Model Parameters
Langmuir isotherm	*q*_max_ = 39.62 (mg g^−1^) *K*_L_ = 0.226 (L mg^−1^) *R*^2^ = 0.98897
Freundlich isotherm	*K*_F_ = 9.54 [mg g^−1^ (L mg^−1^)^n^] 1/*n* = 0.28 *R*^2^ = 0.94305
Temkin	*A*_T_ = 0.37 (L g^−1^) *b*_T_ = 24.46 *B* = 101.29 J mol^−1^ *R*^2^ = 0.97357
Dubinin-Radushkevich	*q*_DR_ = 17.83 (mg g^−1^) *K*_*DR*_ = 1.52 (mol^2^ kJ^−2^) *E* = 0.57 (kJ mol^−1^) *R*^2^ = 0.9497

**Table 4:** Maximum monolayer adsorption capacities (*q*_max_) of various adsorbents used to remove RY145 dye

Adsorbent	*q*_max_ (mg g^−1^)	Reference
Chitosan Functionalized by Talc	76.9	[46]
husk biochar	73	[49]
Chitosan coated magnetic nanoparticles	47.62	[51]
Teff straw-activated carbon	17	[32]
Biochar derived from groundnut Shel	7.33	[33]
NiH/PP microparticles	39.62	This study

Favorable adsorption processes should have *R*_L_ values between zero and one. Values of *R*_L_ greater than one indicate unfavorable adsorption processes [50]. The results *R*_L_ lie between 0.1 and 0.4, which confirms the favorability of the adsorption process (0 < RL < 1). The value of the constant for the heat of sorption (*B*) from the Temkin plot indicates physical adsorption. Additionally, the adsorption energy (*E*) estimated from the Dubinin–Radushkevich isotherm model (Eq. 11) is used to define the type of adsorption: values lower than 8 kJ mol^−1^ indicate a physisorption process, while higher values indicate chemisorption [39]. The value for *E* is well below 8 kJ mol^−1^ (Table 3) revealing that the adsorption process is physisorption.

#### Thermodynamic studies

**Fig. 6:**
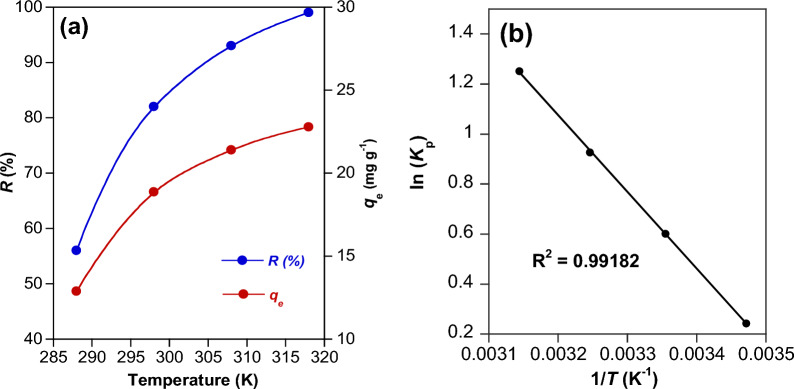
(**a**) Influence of temperature on the adsorption of RY145 dye on NiH/PP microparticles and (**b**) Van’t Hoff plot of ln (*K*_p_) versus (1/T)

Temperature affects the adsorbate diffusion at the adsorbate/adsorbent interface. The impact of temperature on RY145 dye adsorption on NiH/PP microparticles is illustrated in Fig. 6. Both *R* (%) and *q*_e_ of NiH/PP microparticles increased with enhancing temperature (Fig. 6a). The increase in temperature seems to facilitate the movement and dispersion of the adsorbate molecules. This in turn favors the adsorbate/adsorbent interactions, and thus improves the adsorption process. The entropy change (Δ*S*), the enthalpy change (Δ*H*) and the Gibbs free energy change (Δ*G*) of the adsorption process were estimated utilizing the partition coefficient (*K*_p_) (Eqs. 16–19) [52].

16 $$\Delta G = - RTln\left( {K_{p} } \right)$$

17 $$\Delta G = \Delta H - T\Delta S$$

18 $${\text{ln}}\left( {K_{p} } \right) = \frac{\Delta S}{R} - \frac{\Delta H}{R}\frac{1}{T}$$

*K*_p_ can be defined as:

19 $$K_{p} = \frac{{a_{s} }}{{a_{e} }} = \frac{{\gamma_{s} }}{{\gamma_{e} }}\frac{{C_{s} }}{{C_{e} }}$$

where *a*_s_ is the activity of the adsorbed RY 145 dye on NiH/PP microparticles, *a*_e_ is the equilibrium activity of RY 145 dye in solution, γ_s_ and γ_e_ are the activity coefficients of the adsorbed dye on the adsorbent and that in solution at equilibrium, respectively, *C*_s_ and *C*_e_ are the concentrations of adsorbed dye and that in solution at equilibrium (mg L^−1^), respectively. When the concentration of the adsorbate approaches zero (*C*_s_ → 0 and *C*_e_ → 0), the activity coefficient γ approaches unity, and Eq. (19) becomes:

20 $$\mathop {Lim}\limits_{Cs \to 0} \frac{{C_{s} }}{{C_{e} }} = \frac{{a_{s} }}{{a_{e} }} = K_{p}$$

The values of *K*_p_ can be estimated by plotting ln(*C*_s_/*C*_e_) versus *C*_s_ and extrapolating *C*_s_ to zero. The value *K*_p_ is then assessed from the intercept of this straight line. Plotting In (*K*_*P*_) versus (1/*T*) (Eq. 18) yielded a straight line (Fig. 6b), from which the value of Δ*S* is evaluated from the intercept ($\frac{\Delta S}{R}$) and that of Δ*H* is deduced from the slope ($\frac{-\Delta H}{R}$). The thermodynamic parameters (Δ*S*, Δ*H* and Δ*G*) of the adsorption processes are given in Table 5.

**Table 5:** The thermodynamic parameters for the adsorption of RY145 dye on NiH/PP microparticles at different temperatures

*T* (K)	Δ*G* (kJ mol^−1^)	Δ*H* (kJ mol^−1^)	Δ*S* (J mol^−1^ K^−1^)
298	− 1.48	25.52	90.61
318	− 3.29		
323	− 3.75		
333	− 4.65		

Since Δ*H* is positive, the adsorption is considered an endothermic process. The positive value of Δ*S* signifies that the haphazardness increases at the adsorbent/adsorbate interfaces during the adsorption [42]. Δ*G* showed a negative value at 25 °C revealing the spontaneity of the adsorption process even at a low temperature (cost-effective process). The Δ*G* value become more negative with increasing temperature, and thus the viability of the adsorption process can be enhanced by heating [43]. Thus, NiH/PP microparticles show high accessibility for the removal of RY145 dye from aqueous media. Similar thermodynamic observations (Δ*H* and Δ*S* have positive values; Δ*G* has negative values) was reported for uptake of RY145 dye by chitosan functionalized by Talc [46] and husk biochar [49].

#### The adsorption mechanism

**Scheme 1:**
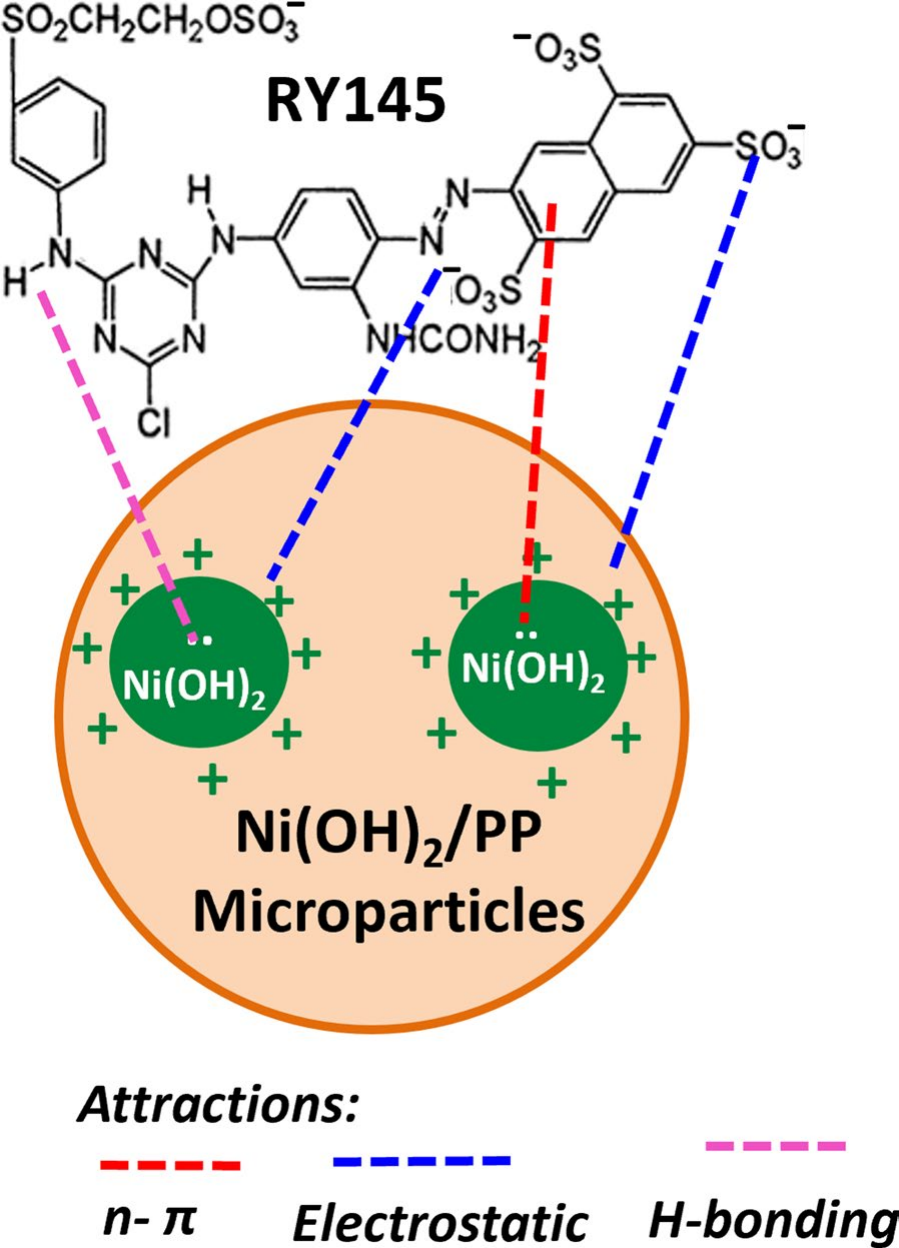
The possible interactions occurred between RY145 dye and NiH/PP microparticles

Hydroxide-containing adsorbents can attract the dye molecules through ion exchange, H-bond formation, electrostatic interactions, π -π, and *n*-π interactions [46, 49]. The pH studies (Fig. 3a) and the FT-IR investigations done on NiH/PP samples covered with the dye (data not shown) confirm the previous observations concerning the adsorption mechanism of RY145 dye on the hydroxide-containing adsorbents. There was no observation for a meaningful alteration in the FTIR spectra of NiH/PP microparticles prior to and after RY145 dye adsorption. This infers that the interactions between RY145 molecules and NiH/PP are mainly electrostatic. pH studies confirmed that the medium pH strongly affected the adsorption process (Fig. 3a). Strongly basic or acidic media affected adversely the firmness of the metal hydroxide, which has an amphoteric nature. In strong acidic media, metal hydroxide tends to solubilize generating metal cations. These cations can combine with the anionic RY145 dye molecules forming insoluble complexes that precipitate from the solution. Alternatively, at moderate pH values, the adsorbent acquires positive charges (because of protonation) or negative charges (through deprotonation). Thus, the protonated adsorbent can effectively uptake the negatively charged molecules of RY145 dye from the medium. Accordingly, the adsorption of RY145 dye on NiH/PP microparticles was dominated by electrostatic attractive forces. Besides, *n*-π interactions could take place between electron pairs on oxygen atoms of the hydroxide groups and the benzene rings present on the dye molecules. H-bonds could also be formed between the hydrogen atoms of the hydroxide and the nitrogen atoms form RY145 dye molecules. The slight rise in the pH of the medium after adsorption reveals the release of hydroxyl groups from the adsorbent. This could a consequence of the anion exchange that may occur between the metal hydroxide and the sulfonate groups on the dye molecules [44]. Therefore, the possible interactions that may occur between NiH/PP microparticles and RY145 dye are the electrostatic attraction, and *n*-π interactions along with ion exchange and H-bonds. These possible interactions are illustrated in Scheme 1.

#### Regeneration studies

**Fig. 7:**
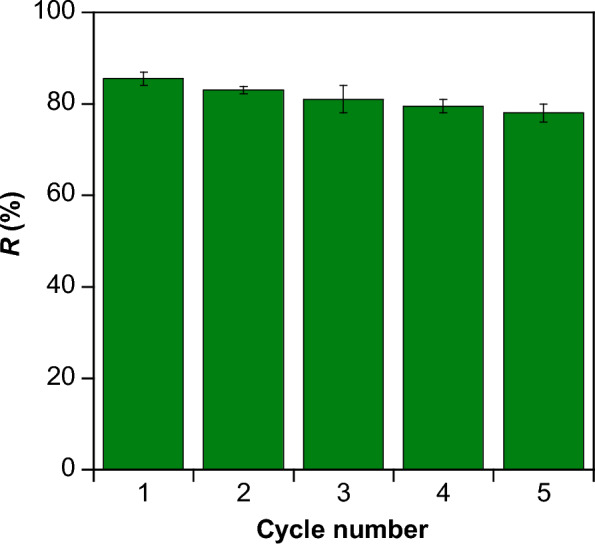
Adsorption cycles performance of NiH/PP microparticles for RY145 adsorption

Studies on the influence of pH on adsorption (Fig. 3 a) show that increasing the pH of the medium reduces the adsorption efficiency of the NiH/PP microparticles. Accordingly, the regeneration of the dye loaded NiH/PP microparticles was carried out at pH = 9, where the adsorbent presumably assumes the deprotonated form that favors the removal of RY145 anionic molecules from its surface. This possibility provides an efficient method to elute RY145 molecules from microparticles, which could then be used for the next adsorption–desorption cycle. Figure 7 shows the results of regeneration and reuse of the adsorbent for five consecutive adsorption/desorption cycles. The removal efficiency of the adsorbent decreases slightly after each adsorption/desorption cycle. However, it is possible to remove 78% of the dye even during the fifth cycle, which reveals reusability of the adsorbent.

## Conclusion

Polypropylene particles were enriched with metal hydroxide microparticles (21.25 wt%) and used as a hybrid adsorbent for removing RY145 dye from water. The structure of the hybrid adsorbent was confirmed by FT-IR and EDS analyses. Batch experiments confirmed that the favorability of the adsorption process can be increased by prolonging contact time, increasing adsorbent dose and heating. However, enhancing the medium pH had a detrimental effect on the adsorption process, showing that dye removal occurs via electrostatic attractions between negatively charged dyes and positively charged NiH/PP. Moreover, ion exchange can also occur between RY145 dye molecules and the hydroxide from NiH/PP microparticles. The experimental adsorption data were best fit with the pseudo-second order kinetic model and the Langmuir isotherm model. The adsorbent reached a maximum adsorption capacity of 39.62 mg g^−1^. The adsorption process was endothermic (∆ *H* =  + 25.52 kJ mol^−1^ K^−1^), random (∆*S* =  +  90.61 J mol^−1^ K^−1^), and spontaneous (∆*G* =  −  1.48 kJ mol^−1^).

## Data Availability

No datasets were generated or analysed during the current study.

## Abbreviations

RY145: Reactive yellow 145
FTIR: Fourier transform infrared spectroscopy
EDS: Energy-dispersive X-ray spectroscopy
SEM: Scanning electron microscopy
∆ *H*: Enthalpy change
∆ *S*: Entropy change
∆ *G*: Free energy change
NiH/PP: Nickel hydroxide/polypropylene
